# Necrosis of the diaphragm pleural empyema and pneumomediastinum as rare complications of appendicitis: A case report

**DOI:** 10.1016/j.idcr.2026.e02571

**Published:** 2026-04-16

**Authors:** Elias Karam, Antoine Legras, Guillaume Brénugat, Nicolas Michot, Martine Ferrandière, Urs Giger-Pabst, Mehdi Ouaïssi

**Affiliations:** a Department of Digestive, Oncological, Endocrine, Hepato-Biliary and Pancreatic Surgery, and Liver Transplantation – Trousseau Hospital, Avenue de la République, Tours F37044, France; b UMR1327 ISCHEMIA, Membrane signaling and inflammation in reperfusion injuries", Université de Tours, 10, Bd Tonnellé, Tours 37000, France; c Department of Vascular, Thoracic and Cardiac Surgery – Trousseau Hospital, Avenue de la République, Tours F37044, France; d Intensive Care Unit, University Hospital of Tours – Trousseau Hospital, Avenue de la République, Tours F37044, France; e Fliedner Fachhochschule, University of Applied Sciences Düsseldorf, Geschwister-Aufricht-Straße 9, Düsseldorf 40489, Germany

**Keywords:** Acute appendicitis, Complication, Empyema, Diaphragm, Necrosis

## Abstract

**Background:**

Appendicitis is one of the most common surgical emergencies in general surgery. To our knowledge, this is the first reported case of diaphragmatic necrosis associated with appendicitis.

**Case:**

A 38-year-old male north African patient with no past medical history presented with fever and right lower quadrant pain, for which he started oral self-medication with ibuprofen. Clinical and radiological examinations revealed perforated appendicitis with right-sided pleural empyema and pneumomediastinum. During the initial surgical procedure, we performed an appendicectomy, inserted a chest tube and administered a broad-spectrum antibiotic therapy. The following day, respiratory failure occurred, necessitating right sided thoracoscopy. This revealed diaphragmatic necrosis, which was treated by excision and tension-free suturing after conversion to open thoracotomy. Six days later, persistent sepsis necessitated a new exploratory laparotomy and a right-sided hemicolectomy with an ileocolostomy to treat colonic necrosis. The postoperative course was uneventful, and the patient was discharged one month after admission. Bowel continuity was restored six months later. After a one-and-a-half-year follow-up, the patient remained healthy with no long-term physical impairment.

**Conclusion:**

To the best of our knowledge, this is the first case of acute appendicitis complicated by diaphragmatic necrosis, empyema and pneumomediastinum to be reported in the medical literature. The pleural empyema and diaphragmatic defect were treated by conversion from thoracoscopy to thoracotomy and direct suture.

## Introduction

Appendicitis is one of the most common abdominal surgical emergencies. It is most common in the second and third decades of life, but it may also affect children under the age of 10 and older adults [1]. The most common presentation is fever and pain in the right iliac fossa. Due to anatomical variations in the appendix location, patient age and potential complications, there is a wide range of possible clinical presentations [2], [3], [4], [5]. Diaphragmatic necrosis and pneumomediastinum are rare complications with only a few cases reported [6], [7], [8], [9], [10], [11], [12], [13]. We present the case of a 38-year-old man with appendicitis complicated by diaphragmatic necrosis.

## Case

A 38-year-old north African male patient with no previous medical history presented to the emergency department with worsening right lower quadrant (RLQ) pain, no fever for one week and preserved bowel function. Prehospital abdominal ultrasound prescribed by his general practitioner (GP) 2 days ago was normal. His wife reported ibuprofen intake for the past 4 days prior to the admission. As his condition worsened, he went straight to the hospital without seeing his GP for a second time.

**Fig. 1:**
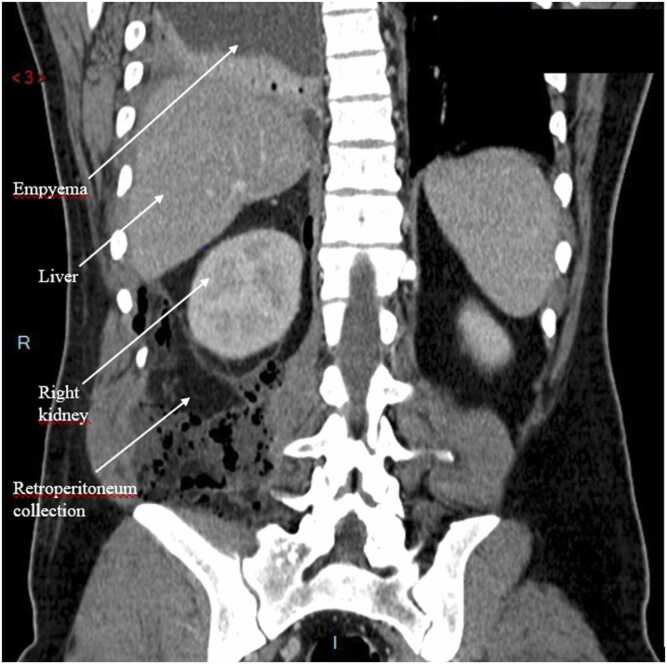
CT scan coronal view of retroperitoneum collection and right-sided empyema.

**Fig. 2:**
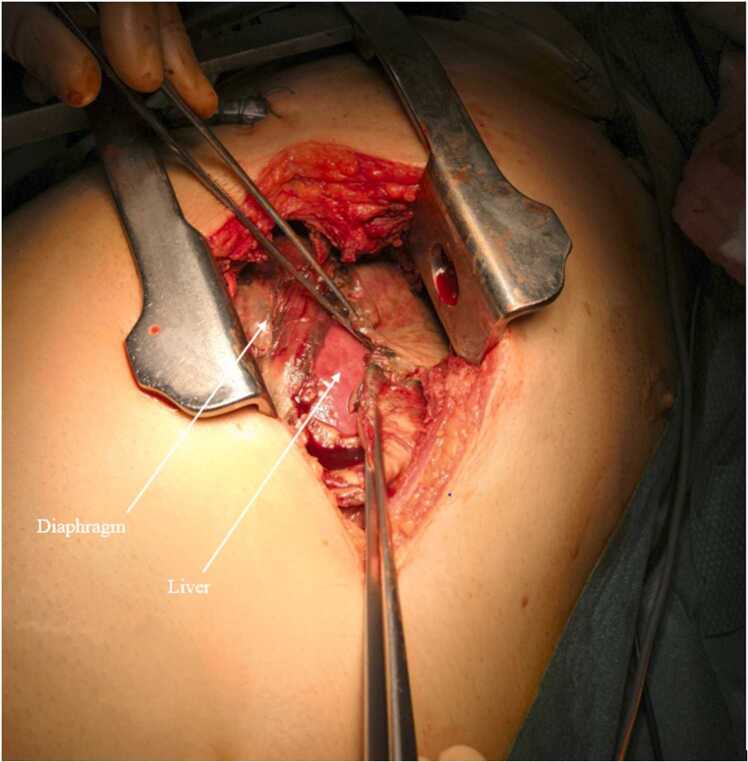
diaphragma necrosis.

**Fig. 3:**
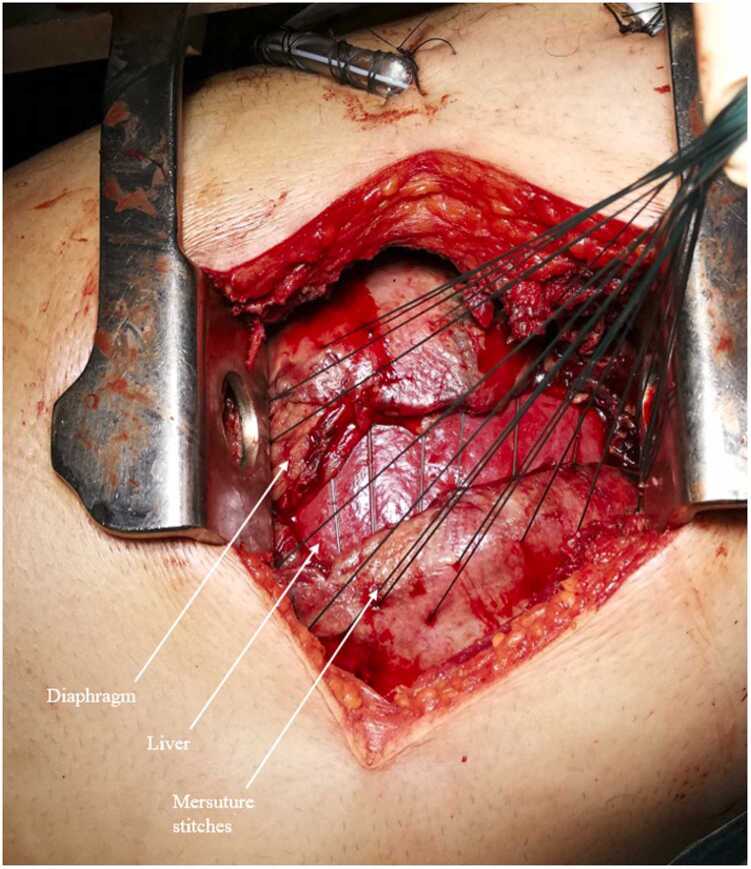
diaphragm separate stitches.

**Fig. 4:**
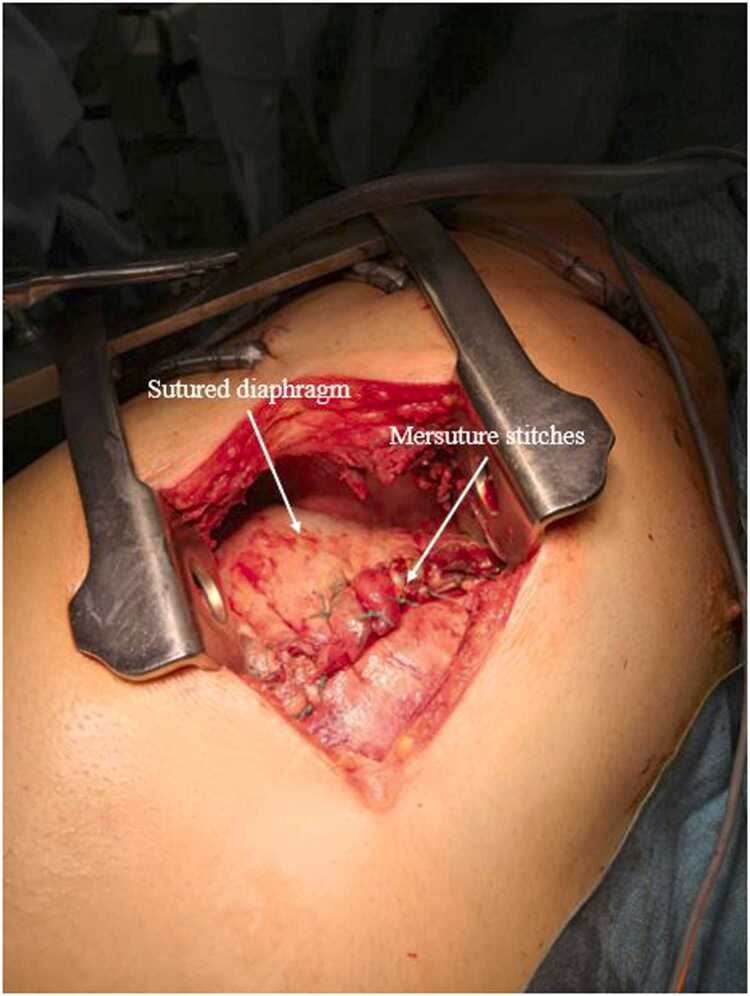
diaphragm suture.

On examination, his vital signs were as follows: blood pressure 140/70 mmHg, heart rate 108 per minute, temperature 38.4°C and oxygen saturation 93% on 6 L of O_2_. Physical examination revealed a grey complexion and tachypnoea. There was no marbling and the RLQ was tender with a non-indurated mass. There was no rebound or guarding. Blood tests showed a white blood cell count of 26.4x10^9^cells/L and a C-reactive protein level of 351.1 mg/L, no coagulation nor liver or renal function test anomaly (*i.e.* prothrombin time 72% activated partial thromboplastin time 16.3, serum alanine transaminase 52IU/L, aspartate aminotransferase 39IU/L, gamma-glutamyl transferase 151IU/L, alkaline phosphatase 39IU/L, bilirubin 11µmol/L, creatinin 115µmol/L). Computed tomography (CT) scan (Fig. 1) showed appendicitis perforated into the retroperitoneum with a 13 mm appendicolith, right sided pleural empyema, and pneumomediastinum. The patient was taken to the operating theatre. Laparoscopic appendicectomy and retroperitoneal drainage were performed along with right-sided thoracic drainage, which yielded 500 ml of pus. The patient was then admitted to the intensive care unit (ICU) and needed vasopressor administration (norepinephrine) initiated during surgery over the septic shock. On day 1, videothoracoscopy was performed to investigate respiratory failure, followed by thoracotomy due to a 5x12cm area of diaphragmatic necrosis (Fig. 2). Necrotic tissue was excised, and the diaphragm was closed with a direct tension-free suture; thoracic drainage was also performed (Fig. 3, Fig. 4). On day 6, a CT scan to explore persistent fever and abdominal pain revealed right colonic necrosis treated with an open right hemicolectomy and ileocolostomy. On day 9, recurrent respiratory failure with a subdiaphragmatic collection on CT scan led to redo thoracotomy. Another diaphragmatic necrotic area measuring 5x5cm was removed and the defect was closed with direct tension-free suture. Samples from the abdomen and thorax revealed *Escherichia coli, Streptococcus anginosus, Enterococcus faecium, Citrobacter koseri, Bacteroides fragilis, Bacteroides thetaiomicron*, and *Pseudomonas aeruginosa*. The same germs were retrieved in the repeated blood cultures. The initial antibiotic therapy of CEFTRIAXONE/METRONIDAZOLE/GENTAMICIN was changed to PIPERACILLIN-TAZOBACTAM/METRONIDAZOLE, then to IMIPENEM-CILASTATIN/METRONIDAZOLE, and finally to MEROPENEM/METRONIDAZOLE, until discharge. There were no further complications, so care focused primarily on physical therapy, functional rehabilitation, respiratory rehabilitation, and stoma education. The patient was discharged from the ICU 10 days later after removal of all drains. He was discharged from the hospital one month after being admitted. The pathology report confirmed perforated phlegmonous appendicitis and transmural necrosis of the removed diaphragm and right colon. Bowel continuity was restored six months later. Surgical follow-up was performed every three months, until one and a half years after the index surgery with clinical and radiological examinations. There were no long-term clinical impairments, and the patient had a normal quality of life.

## Discussion

This case describes a perforated appendicitis complicated by diaphragmatic necrosis that required multiple surgical procedures. The patient had abdominal pain for one week and had taken ibuprofen, despite having no previous medical history. Thoracic empyema is a rare complication of appendicitis. Vasquez-Rios and colleagues conducted in 2018 a literature review of all 10 published case reports of empyema complicating appendicitis [6]. Patterns in these cases suggested that it is more common in children [11], the elderly [12] and pregnant women [13]. All patients survived. Escherichia coli, Streptococcus spp, and Bacteroides spp were the most commonly identified pathogens. Pneumomediastinum is also a rare complication of appendicitis, with only three cases reported in the literature [8], [9], [10] and we found several cases associated with necrotising fasciitis and/or colitis [14], [15], [16]. The physiopathology is unclear, but some authors suggested lymphatic spread of infection, anatomical communication between the thoracic and abdominal cavities [17], or diaphragm erosion by stercolith migration. In our case, the mechanism may have involved a combination of factors: contiguous infection of the surrounding tissues; spread of air and infection from perforated appendicitis to the retroperitoneum and mediastinum, later exacerbated by sepsis, hypoperfusion and norepinephrin administration. Iatrogenic drainage-related diaphragm perforation may be involved either as primary (*i.e.* traumatic perforation) or secondary cause (*i.e.* unveiling a preexisting sealed perforation). In this case, the large necrotic diaphragmatic area suggested primary inflammatory/vascular cause however we cannot exclude that the first chest drainage may have perforated a fragilized diaphragm. As this drainage was performed under general anaesthesia and videothoracoscopy the day after, the main worrisome clinical feature was the persisting respiratory failure. Pulmonary embolism or iatrogenic causes (*i.e.* ICU ventilation settings) may participate but were not reported in this case. This occurred in a patient with a delayed diagnosis explained by the atypical clinical presentation and the initially normal ultrasound findings. In addition to the patient’s age, the atypical clinical presentation may also be related to ibuprofen intake, as non-steroidal anti-inflammatory drugs are known to worsen infections while masking their symptoms, particularly pain [18]. Ultrasound imaging is readily available, especially outside of hospitals. However, its performance depends on the operator and is inferior to that of CT scans for diagnosing complicated appendicitis [19]. CT scan with contrast injection is mandatory to exclude differential diagnoses such as subphrenic or pulmonary abscess, pneumonia or pleural effusion.

## Conclusion

Diaphragmatic necrosis is a rare but possible complication of appendicitis whose physiopathology remains unclear. Atypical clinical presentations such as associated respiratory failure and/or persistent sepsis after appendectomy may guide the diagnosis. Surgical management involving both visceral and thoracic surgeons is required.

## CRediT authorship contribution statement

**Urs Giger-Pabst:** Writing – review & editing. **Martine Ferrandière:** Writing – review & editing. **Antoine Legras:** Writing – review & editing. **Ouaïssi Mehdi:** Writing – review & editing, Writing – original draft, Data curation, Conceptualization. **Nicolas Michot:** Writing – review & editing, Writing – original draft, Data curation, Conceptualization. **Elias Karam:** Writing – review & editing, Writing – original draft, Data curation, Conceptualization. **Guillaume Brénugat:** Writing – review & editing

## Funding

This research did not receive any specific grant from funding agencies in the public, commercial, or not-for-profit sectors.

## Declaration of Competing Interest

The authors declare that they have no competing interests
